# Supplementary material for: Therapeutic Effect of a Latent Form of Cortistatin in Experimental Inflammatory and Fibrotic Disorders
Source: Pharmaceutics. 2022 Dec 13;14(12):2785. doi: 10.3390/pharmaceutics14122785 (PMC9784182; doi:10.3390/pharmaceutics14122785)
Supplement: Supplementary file 1 [file pharmaceutics-14-02785-s001.zip › pharmaceutics-2000498-supplementary.pdf]

Article

Therapeutic Effect of a Latent Form of Cortistatin in Experimental Inflammatory and Fibrotic Disorders

Jenny Campos-Salinas *, Margarita Barriga and Mario Delgado *

Supplementary Figures

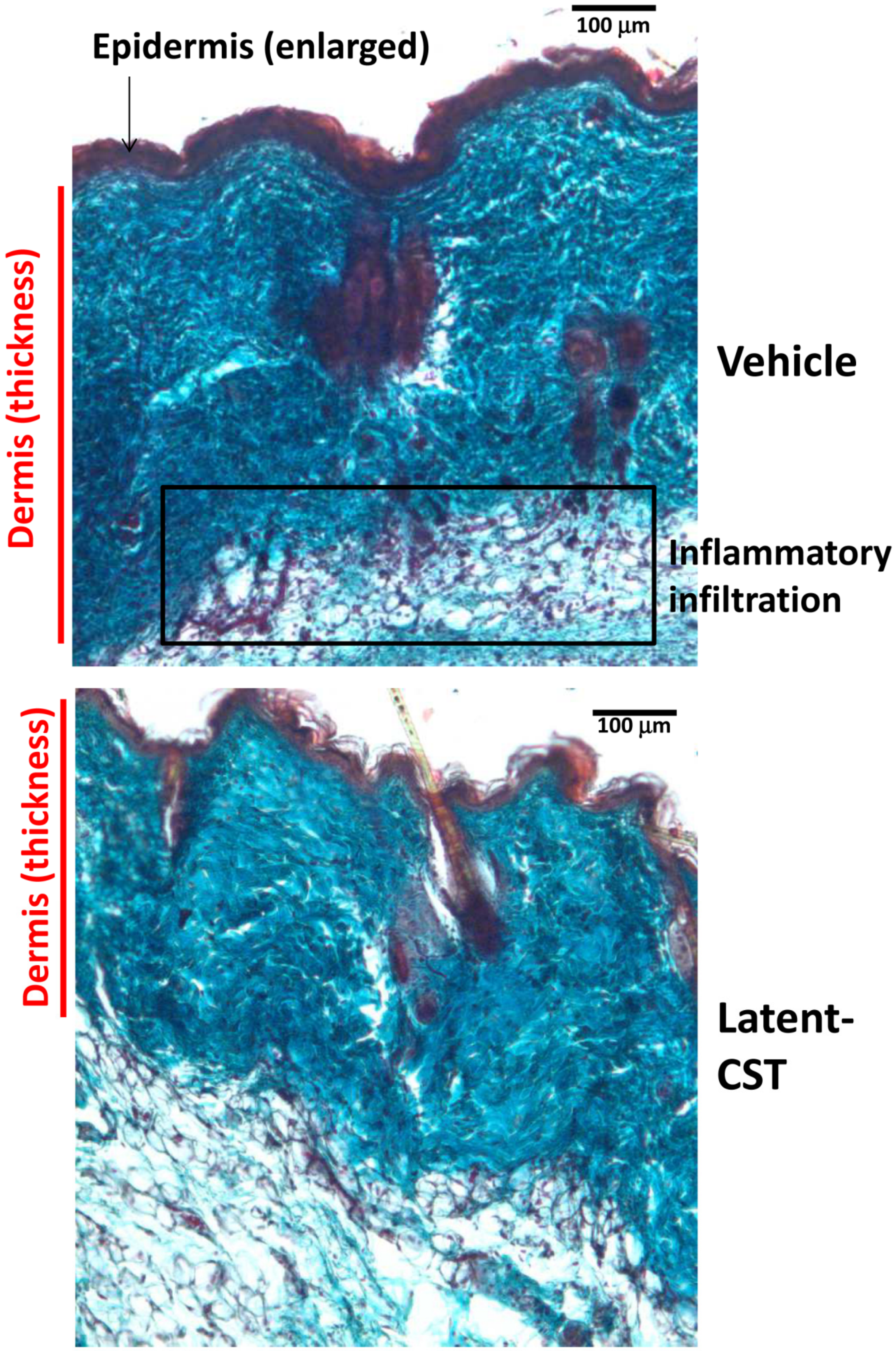

Supplementary Figure S1. Histological sections of skin biopsies from mice with bleomycin-induced dermal fibrosis (see Figure 5) showing dermal thickness, enlargement of epidermal layer and inflammatory infiltration (square in hypodermis) in vehicle-treated animals. Latent-CST treatment reduced these histopathological signs.

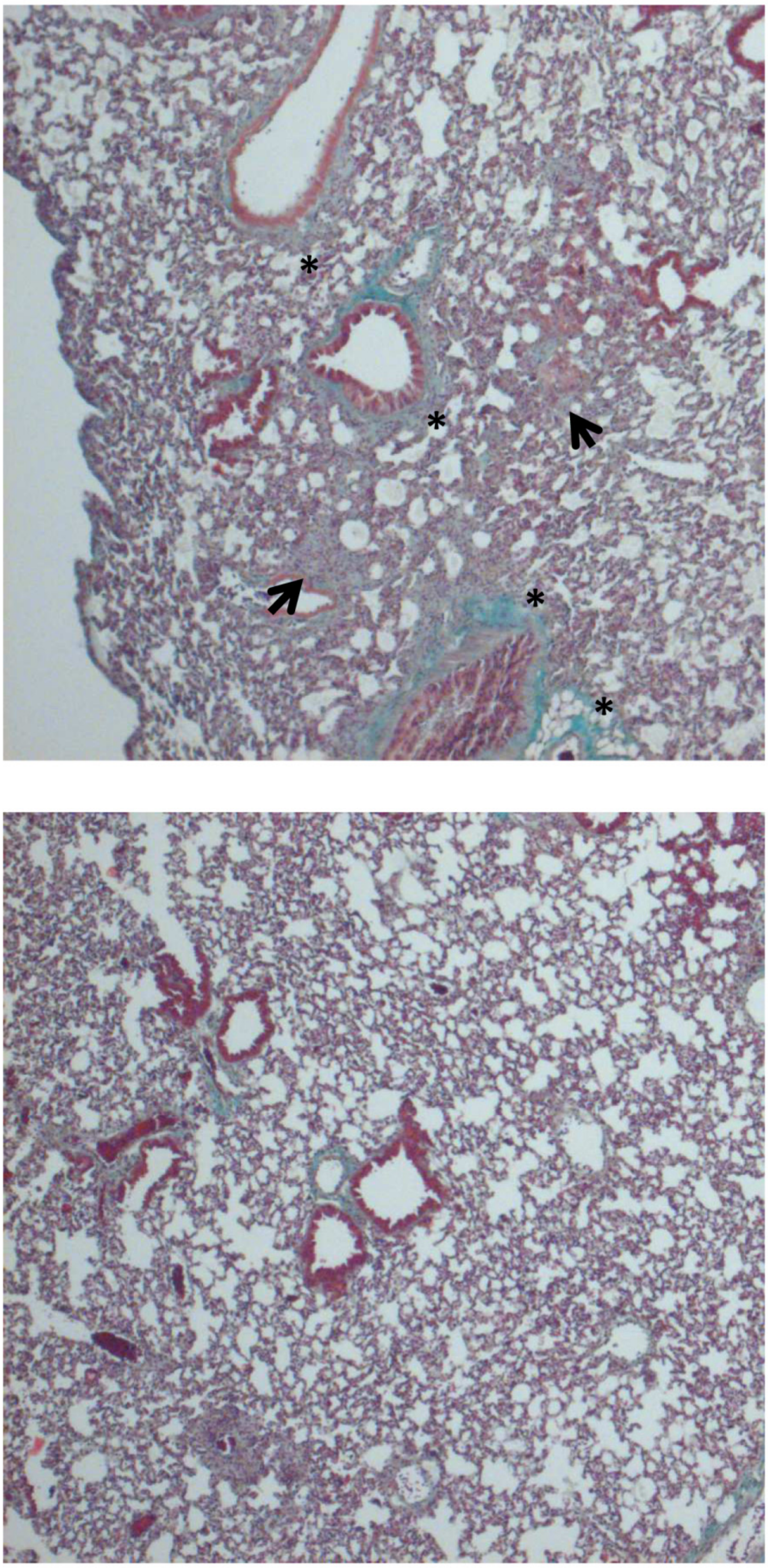

Vehicle

Latent-CST

Supplementary Figure S2. Histological sections of lung biopsies from mice with bleomycin-induced dermal fibrosis (see Figure 5) showing marked pulmonary fibrosis (asterisks), generalized thickening of alveolar septa and foci with almost devoid of alveolar space (arrows) in the vehicle-treated animals. Latent-CST treatment significantly avoided these histopathological signs.

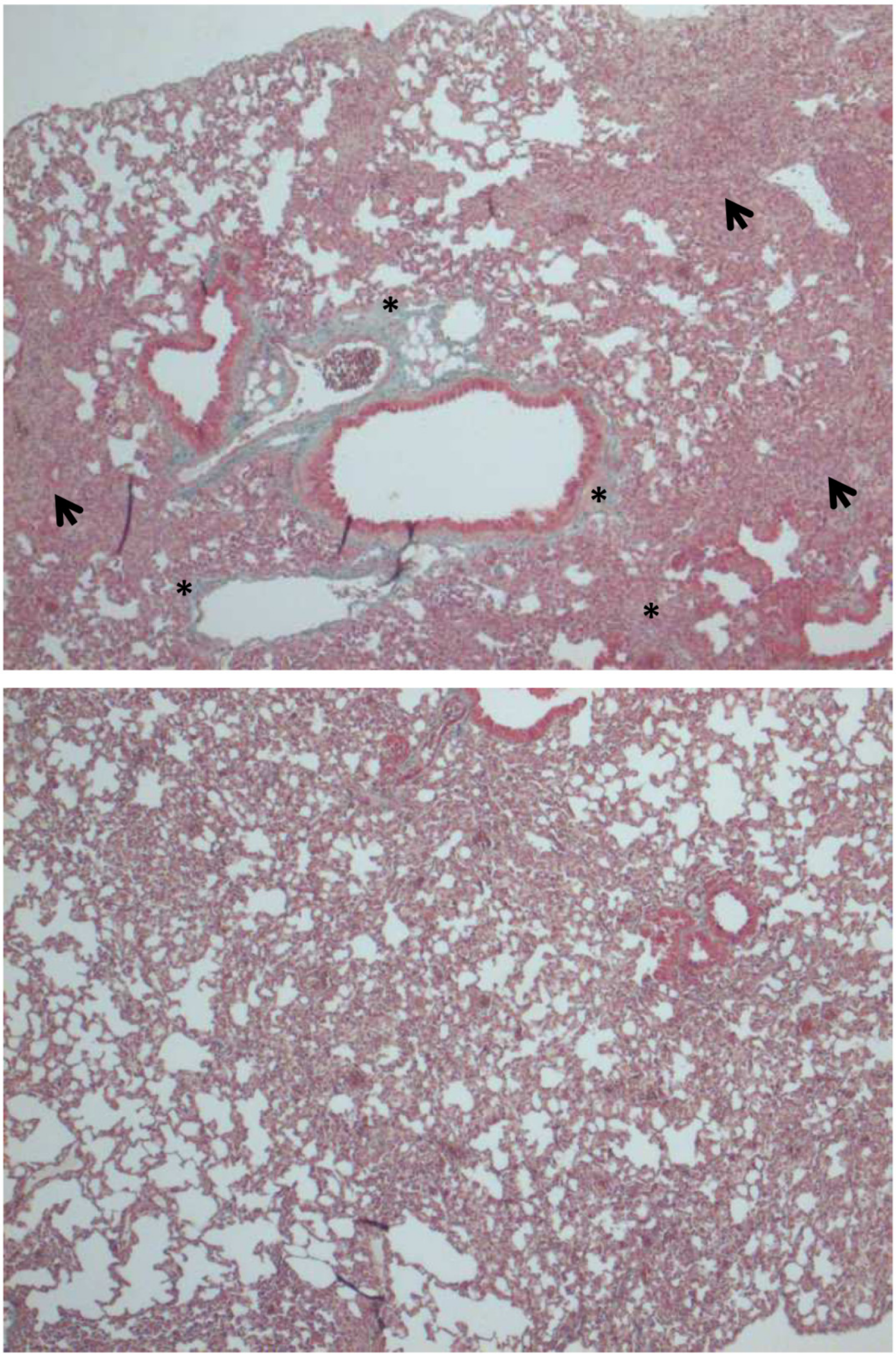

Vehicle

Latent-CST

Supplementary Figure S3. Histological sections of lung biopsies from mice with bleomycin-induced pulmonary fibrosis (see Figure 6b-c) showing extensive peribronchiolar fibrosis (asterisks), marked damage of pulmonary architecture with significant thickening of alveolar septa and extensive areas with obliterated alveolar space (arrows) in the vehicle-treated animals, which were significantly reduced by the treatment with Lantent-CST.
